# The Diseases to Which the Domestic Animals Are Most Subject

**Published:** 1882-04

**Authors:** 


THE DISEASES TO WHICH THE DOMESTIC ANIMALS ARE MOST SUBJECT.—Some interesting facts in regard to the diseases from which the domestic animals suffer most are given by Dr. A. Lydtin, in his report as Superintendent of Veterinary State Medicine, in the Grand Duchy of Baden.

There were treated in the year 1881, in this Duchy, 22,000 animals. The per cent. of cases treated were as follows: Cattle, 54; horses, 37; sheep, 3; dogs, 3; swine, 2; goats, 1.

The kinds of diseases from which these animals suffered were, in one hundred cases, as follows: Digestive organs, 29 per cent.; respiratory organs, 13 per cent.; constitutional diseases (fevers, etc.), 13 per cent.; surgical operations (for injuries, but chiefly castrations), 13 per cent.; external diseases (skin, etc.), 12 per cent.; diseases of the organs of locomotion (rheumatism, lameness, etc.), 10 per cent.; diseases of the nervous system, 3 per cent.; diseases of the kidneys, 2 per cent.; parasitic diseases, 2 per cent.; localized disease 1 per cent.; circulatory diseases, 1 per cent.; infectious diseases, 1 per cent.

This list shows a comparatively small proportion of diseases among horses for which treatment is required. Also, a much smaller number of cases of lameness than would be found in large cities. The proportion given, however, will probably hold good for the country and large towns, where there is no epidemic disease of any kind.

The animals were treated by 106 veterinary surgeons, making an average of over two hundred cases for each practitioner.

